# Electroacupuncture versus sham for insomnia in patients undergoing maintenance hemodialysis: study protocol for a pilot randomized controlled trial

**DOI:** 10.1080/07853890.2025.2496410

**Published:** 2025-04-29

**Authors:** Anming Lu, Mina Wang, Nan Jiang, Huilin Liu, Ying Gao, Tong Wang, Xiaobai Xu, Bin Li

**Affiliations:** ^a^ Department of Nephrology and Rheumatology, Beijing Dongcheng First People’s Hospital, Beijing, China; ^b^ Graduate School, Beijing University of Chinese Medicine, Beijing, China; ^c^ Department of Acupuncture and Moxibustion, Beijing Hospital of Traditional Chinese Medicine, Capital Medical University, Beijing Key Laboratory of Acupuncture Neuromodulation, Beijing, China

**Keywords:** Insomnia, end-stage renal disease, maintenance hemodialysis, electroacupuncture, sham electroacupuncture

## Abstract

**Background:**

Insomnia causes severe health problems and poor quality of life in patients with end-stage renal disease (ESRD) undergoing maintenance hemodialysis (MHD). Conventional therapies are limited due to the poor availability and opaque evidence, and electroacupuncture (EA) is considered an alternative for improving insomnia in patients with ESRD undergoing MHD.

**Methods:**

This is a study protocol for a pilot randomized, sham-controlled, single-center, clinical trial. A total of 60 patients with insomnia in ESRD undergoing MHD will be randomly assigned to treatment group (EA, 3 sessions per week for 4 weeks), or sham group (sham EA, 3 sessions per week for 4 weeks) in a 1:1 ratio. Participants will complete the trial by visiting the research center at Week 8 for a follow-up assessment. The primary outcome is the response rate: the proportion of patients achieving a minimal clinically important difference, which is defined as ≥7 points on insomnia severity index (ISI) at Week 4 compared with baseline. Secondary outcomes include sleep diary, ISI, pittsburgh sleep quality index (PSQI), short form health survey-12 (SF-12), fatigue severity scale (FSS), 24-Hour ambulatory blood pressure, and plasma levels of C-reactive protein (CRP) and D-Dimer.

**Discussion:**

This is the first standardized protocol investigating the efficacy and safety of EA for patients with ESRD undergoing MHD. This trial may provide evidence for clinical application of EA for patients with ESRD undergoing MHD.

**Trial Registration:**

Chinese Clinical Trial Registry (registered number: ChiCTR2300071814).

## 1. Introduction

Insomnia is defined as persistent difficulty in falling or maintaining sleep or waking up too early despite adequate sleeping opportunities and circumstances for sleep, which produces impairment of daytime function. Chronic insomnia is characterized by sleeping disturbances occurring at least three times per week for at least 3 months [1].

Insomnia is one of the most common problems in patients with all stages of kidney dysfunction, and worsening chronic kidney disease (CKD) or end-stage renal disease (ESRD) is directly associated with worsening insomnia [2], which results in fatigue, impaired circadian rhythm, poor overall quality of life [3], and increased complications such as depression, impaired immune response [4]. Insomnia occurs in more than half of patients with predialysis CKD, with a prevalence of up to 69% in patients with ESKD [5]. Insomnia in ESRD patients is affected by a variety of factors, including elderly age, dialysis vintage and a higher parathyroid hormone level [6], in which, patients treated with maintenance hemodialysis (MHD) have ranked insomnia as one of the major issues that reduce their quality of life [5]. Studies have suggested that 40% to 85% of patients undergoing dialysis have sleep disturbances [7], and a study conducted in the US demonstrated that among 1,643 patients from 335 US dialysis centers, 50% had difficulty falling asleep, 59% woke during the night, and 49% woke up too early; 53% had one or more of these symptoms all or most of the time [8]. Insomnia in ESRD patients increases nocturnal systolic blood pressure variability (BPV) [9] and inflammatory factors such as high-sensitivity C-reactive protein (hsCRP), interleukin 1β (IL-1β) [10], which might contribute to disease progression and worsen prognosis. A study has demonstrated that poor sleep quality reduced patient’s quality of life and triggered higher mortality [11].

Currently, there is no specific nonpharmacologic or pharmacologic recommendation for insomnia management in MHD patients, the American College of Physicians recommends cognitive behavioral therapy for insomnia (CBT-I) as the first-line treatment for insomnia, including stimulus control, sleep restriction and consolidation, cognitive restructuring, and sleep hygiene [12,13]. However, there are few physicians trained in CBT-I and CBT-I will be an extra treatment burden for patients with MHD who already have extremely hectic treatment schedule. Therefore, CBT-I should be adjusted particularly for these patients, and physicians need to be further trained based on the unique circumstances of these individuals. Except for CBT-I, pharmacotherapy is commonly used in treating insomnia, whereas there is no consensus about which sleep medication should be recommended to MHD patients due to the limited evidence about efficacy and safety [3,7]. Although benzodiazepines and nonbenzodiazepine receptor agonists are often prescribed for MHD patients, attention should also be paid to the risk of side effects and death in MHD patients [14], for example, benzodiazepines may cause Alzheimer’s disease or stroke [15,16]. Besides, many physicians are reluctant to prescribe sleep medications for MHD patients because of the limited efficacy and safety data. Therefore, most patients with ESKD undergoing MHD who have insomnia do not receive any treatment, which causes a vicious cycle for them.

Alternative treatment such as acupuncture has been recognized with the efficacy and safety in treating insomnia [17]. A clinical trial has suggested electroacupuncture (EA) significantly improves the quality of sleep in patients with depression [18]. Moreover, acupuncture promotes renal function, reduces proteinuria, controls hypertension, corrects anemia, relieves pain, and controls many hemodialysis-related complications including insomnia in patients with CKD [19,20]. However, there is no clinical trial demonstrating the efficacy and safety of EA in treating insomnia in ESRD undergoing MHD, and a sham-controlled trial is considered by the systematic review to have a low risk of bias in all areas, including selection, conduct, detection, attrition and reporting [21]. Therefore, our study aims to investigate the efficacy and safety of EA in relieving insomnia in ESRD undergoing MHD using a randomized, sham-controlled clinical trial. We present this article in accordance with the SPIRIT reporting checklist.

## 2. Methods/design

### 2.1. Study design and setting

**Figure 1.**
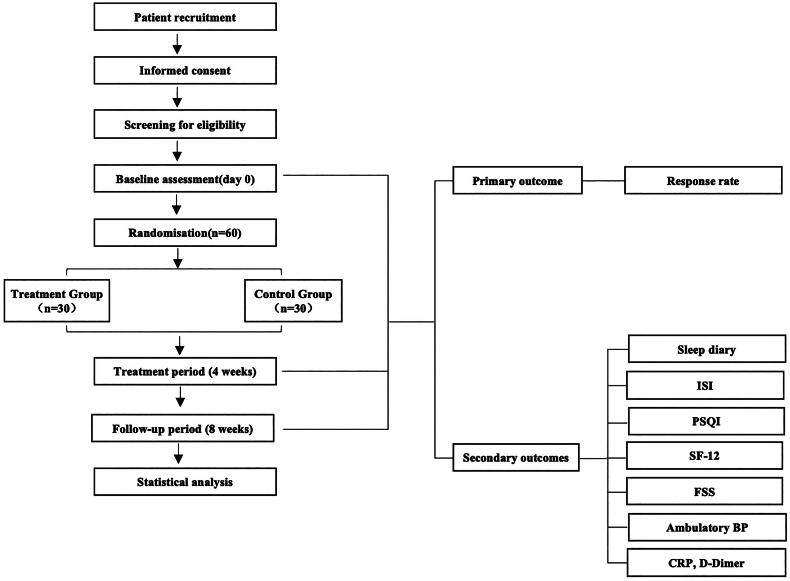
Flow diagram. BP: blood pressure, CRP: C-reactive protein, FSS: fatigue severity scale ISI: insomnia severity index, PSQI: Pittsburgh sleep quality index, SF-12: short form health survey-12.

**Figure 2.**
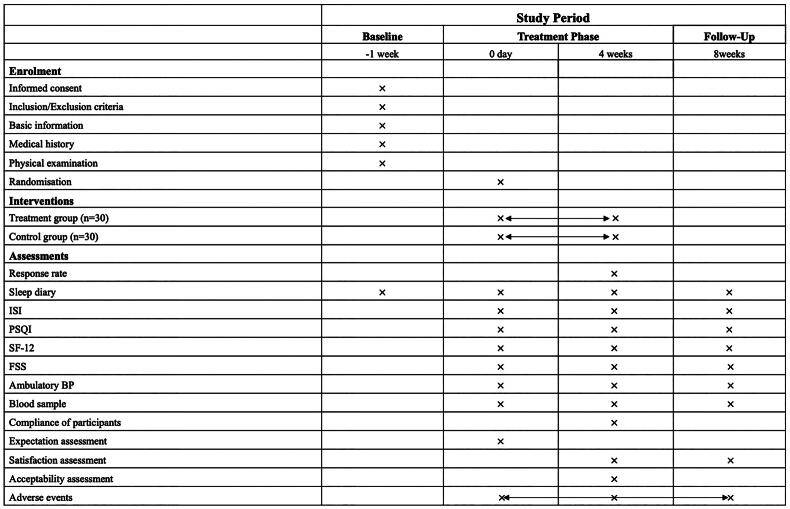
Schedule for data collection of this trial. X: the moment of enrolment, intervention or assessments. Double-headed arrows: the whole phase of intervention or adverse events assessment. BP: blood pressure, CRP: C-reactive protein, FSS: fatigue severity scale ISI: insomnia severity index, PSQI: Pittsburgh sleep quality index, SF-12: short form health survey-12.

This study proposes a single-center, sham-controlled, randomized controlled trial (RCT) at Beijing Dongcheng First People’s Hospital. The study was approved by the research ethic committee. The study will undergo quarterly audits by the Ethics Committee to determine if early termination is necessary. This study will be conducted following the Declaration of Helsinki (as revised in 2013). Written informed consent will be obtained from all participants. Recruitment strategies include posting online advertisement *via* the hospital website and Chinese social media (WeChat), as well as flyers at outpatient unit [22,23]. The intervention includes 4 weeks of EA or sham EA and 4 weeks of follow-up (Figures 1 and 2).

### 2.2. Participants

Participants will be recruited from the hemodialysis department of Beijing Dongcheng First People’s Hospital. The enrolment began in June 2023 and is anticipated to be completed by June 2024. Each eligible and consented patient will be randomly assigned in a 1:1 ratio to receive 4-week EA or sham EA.

#### 2.2.1. Diagnosis criteria

End-stage renal disease will be diagnosed based on ‘Practice Guideline for the Evaluation and Management of Chronic Kidney Disease (2012 Edition)’ [24]. Stage 5 chronic kidney disease or requirement of maintenance hemodialysis will be diagnosed based on ‘Guidelines for early screening, diagnosis, prevention and treatment of chronic kidney disease (2022 Edition)’ [25]. Insomnia will be diagnosed based on ‘Guidelines for diagnosis, treatment of insomnia in Chinese adults (2017 Edition)’ [26].

#### 2.2.2. Inclusion criteria

1. Aged between 18 and 75 years, male or female
2. Diagnosed with end-stage renal disease and chronic insomnia based on the abovementioned diagnosis criteria
3. Insomnia Severity Index (ISI) ≥15
4. Maintenance hemodialysis treatment ≥3 months with stable condition
5. Agreement of using zopiclone as rescue medication
6. Provision of written informed consent

#### 2.2.3. Exclusion criteria

1. Renal transplant patients
2. Having hemorrhagic disease, neurological disease, metabolic disease, hematopoietic system disease, infectious disease or other severe acute or chronic organic diseases
3. History of long-term use of sleeping pills before hemodialysis treatment
4. Having taken sleeping pills within 2 weeks before the first hemodialysis treatment
5. Having unstable uremia complications such as water-electrolyte and acid-base disturbances, calcium and phosphorus metabolism disorders, cardiac insufficiency
6. Having acupuncture therapy in the last 3 months
7. Having mental disorders or severe cognitive disorders
8. Pregnant or breastfeeding women
9. Having taken part in other clinical trials within the past 3 months
10. Unable to cooperate or unwilling to comply with all study requirements
11. With cardiac pacemaker, metal allergy, or needle phobia

### 2.3. Randomization and allocation concealment

Participants will be randomly allocated in a 1:1 ratio to either the treatment group (EA) or control group (sham EA). An independent statistician, who will not be involved in the later implementation or statistical analysis, will generate the randomization sequence using block randomization with a block size of 6 or 4 *via* R software (4.2.1). The enrollment number will be written on the cover of an opaque envelope where the randomization sequence will be placed. The Research Center of Clinical Epidemiology (Beijing Institute of Traditional Chinese Medicine) will provide a randomization sequence and opaque envelopes. Researchers will be required to open the envelope according to the enrollment order of participants and apply the treatment instructions specified for their respective groups [22].

### 2.4. Blinding

Throughout the trial, participants will be blinded, however, acupuncturists will not be blinded due to the nature of the intervention. Blinding of group assignments will also be enforced for data analysts and outcome assessors. The concealment of the allocation for all participants will be maintained until the statistical analysis has been completed.

### 2.5. Interventions

The style of acupuncture is based on traditional Chinese medicine principle, and acupuncture prescription has been developed from literature review [27] as well as the clinical practice. Each participant will be ushered by a research assistant to hospital bed, which will be screened off separately one-by-one by opaque curtains. No companion will be allowed to stay with the participants during the treatment period. Acupuncturists should have a Chinese medicine practitioner license with at least 2 years of experience, and two acupuncturists will be given trial-specific training, such as the selection of acupuncture points and the usage of EA apparatus in different groups at the program launching session to realize their role and job in this study. Each participant will be performed EA or sham EA by the same acupuncturist throughout the trial. Also, two acupuncturists will not tell anyone else about the treatment allocation, and they will be asked to communicate with participants as less as possible to avoid inadvertent unblinding.

Sterile disposable acupuncture needles (length, 25–40 mm; diameter, 0.25–0.30 mm; Hwato Brand, Suzhou Medical Appliance Factory, Suzhou, China) and EA apparatus (HANS-200A acupoint nerve stimulator, Nanjing Jisheng Medical Co, Ltd) will be used in both groups. EA apparatus will be connected right after needles are inserted into all acupuncture points, and will be retained for 30 min. All participants will receive the same treatment according to their group. Both the treatment and sham groups will consist of 12-session intervention over 4 weeks (three sessions per week, ideally every other day). Intervention will be discontinued if the participants have any serious adverse events. All participants will be informed that the current of EA may not be perceived by human.

MHD treatment will be applied using sodium bicarbonate dialysate, dialyser (Braun Co, Ltd or Fresenius Co, Ltd, German), and disposable dialyzer membranes (diacetate membrane or polysulfone membrane) in both groups. The dialysate flow rate will be 500 ml/min and blood flow rate will be 200–250ml/min. The frequency will be 3 times per week with 3.5 to 4-h duration each time. The vascular access includes arteriovenous fistula (AVF), arteriovenous graft (AVG), long-term central vein catheter (CVC), and temporary CVC. Unfractionated heparin (UFH) or low-molecular-weight heparin (LMWH) will be applied as an anticoagulant to all participants. Other supportive interventions include treatments for underlying diseases and complications such as hypertension, diabetes, infections, renal anemia, and water and electrolyte metabolism disorders.

#### 2.5.1. Treatment group

**Figure 3.**
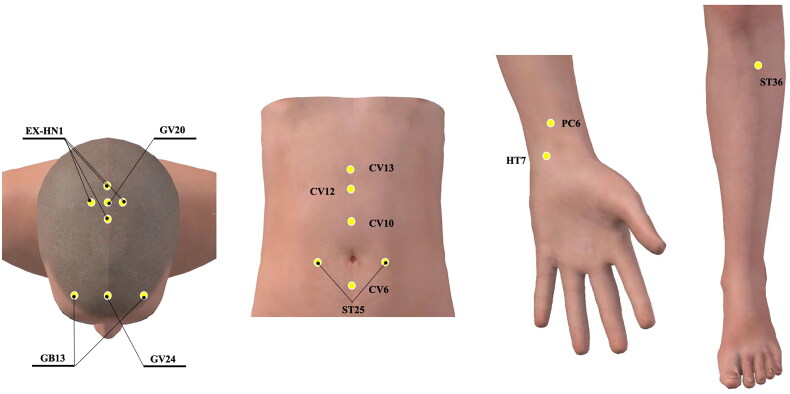
Locations of acupuncture points used in electroacupuncture group. EX-HN1: Sishencong, GV20: Baihui, GB13: Benshen, GV24: Shenting, CV13: Shangwan, CV12: Zhongwan, CV10: Xiawan, CV6: Qihai, ST25: Tianshu, PC6: Neiguan, HT7: Shenmen, ST36: Zusanli.

Acupoints of Baihui (GV20), Shenting (GV24), Sishencong (EX-HN1), bilateral Benshen (GB13), Shangwan (CV13), Zhongwan (CV12), Xiawan (CV10), Qihai (CV6), bilateral Tianshu (ST25), bilateral Neiguan (PC6), bilateral Shenmen (HT7), and bilateral Zusanli (ST36) will be used in EA group (locations are shown in Table 1 and Figure 3). With participants in a prone position, acupuncturists will use 75% alcohol pads to sterilize the skin around the acupoints and then insert disposable needles into the acupoints. For Baihui (GV20), Shenting (GV24), Sishencong (EX-HN1), bilateral Benshen (GB13), the needles will be inserted approximately 5–10 mm with a 30° angle between the needle tip and the scalp, for Shangwan (CV13), Zhongwan (CV12), Xiawan (CV10), Qihai (CV6), bilateral Tianshu (ST25), bilateral Neiguan (PC6), bilateral Shenmen (HT7), and bilateral Zusanli (ST36). Needles will be inserted at an angle of 90° and a depth of 20–30 mm. Lifting and thrusting combined with twirling and rotating will be performed for at least 10 s per acupoint to reach the De qi sensation (soreness, numbness, distention, and heaviness). Then a pair of electrodes from the EA apparatus will be connected to the needle handles at Baihui (GV20), Shenting (GV24). The EA waveform adopts 2/100 hz density alternating wave and the intensity of electric current will be regulated until the participants can perceive and tolerate it. Immediately after needle removal, acupuncturists will gently press acupoints with dry sterilized cotton balls to avoid bleeding.

**Table 1.** Locations of acupuncture points used in electroacupuncture group.

Acupoints	Location
GV20	On the midline of the head, 7 cun directly above the posterior hairline, approximately on the midpoint of the line connecting the apexes of the two auricles.
EX-HN1	A group of four points, at the vertex, 1 cun respectively posterior, anterior, and lateral to GV 20.
GV24	0.5 cun directly above the midpoint of the anterior hairline.
GB13	0.5 cun within the hairline of the forehead, 3 cun lateral to GV 24.
CV6	On the midline of the abdomen, 1.5 cun below the umbilicus.
CV10	On the midline of the abdomen, 2 cun above the umbilicus.
CV12	On the midline of the abdomen, 4 cun above the umbilicus.
CV13	On the midline of the abdomen, 5 cun above the umbilicus.
ST25	2 cun lateral to the center of the umbilicus.
PC6	2 cun above the transverse crease of the wrist, between the tendons of muscle palmaris longus and muscle flexor radialis.
HT7	At the ulnar end of the transverse crease of the wrist, in the depression on the radial side of the tendon of muscle flexor carpi ulnaris.
ST36	3 cun below ST 35, one finger breadth from the anterior crest of the tibia, in muscle tibialis anterior.

GV20: Baihui, EX-HN1: Sishencong, GV24: Shenting, GB13: Benshen, CV6: Qihai, CV10: Xiawan, CV12: Zhongwan, CV13: Shangwan, ST25: Tianshu, PC6: Neiguan, HT7: Shenmen, ST36: Zusanli.

#### 2.5.2. Sham group

**Figure 4.**
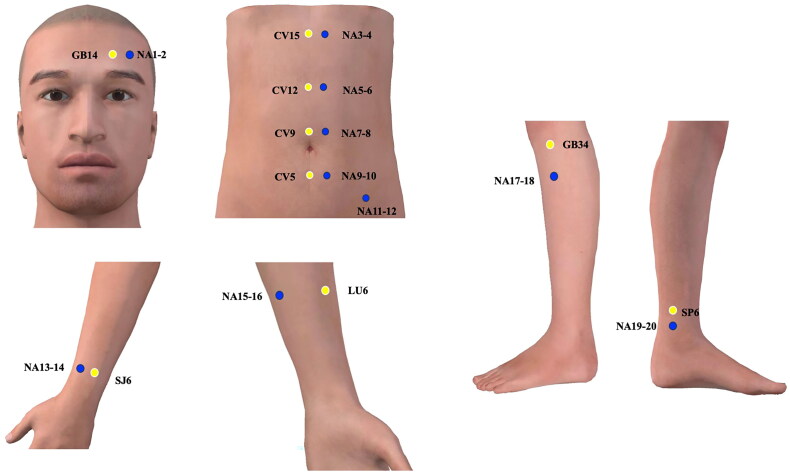
Locations of acupuncture points used in sham electroacupuncture group. Yellow dots: acupoints used for locating non-acupoints; blue dots: non-acupoints. NA: Non-acupoint, GB14: Yangbai, SJ6: Waiguan, CV15: Jiuwei, CV12: Zhongwan, CV9: Shuifen, CV5: Shimen, LU6: Kongzui, GB34: Yanglingquan, SP6: Sanyinjiao.

Twenty non-acupoints based on literature review [28,29] and the clinical practice will be used in sham group (locations are shown in Table 2 and Figure 4). Participants will use an opaque patch to cover their eyes and lie in a prone position, acupuncturists will use 75% alcohol pads to sterilize the skin around the acupoints and then insert disposable needles into the acupoints. Needles will be inserted at an angle of 90° and a depth of 2–3 mm (superficial penetration) in acupoints located in face and will be inserted at an angle of 90° and a depth of 2–3 mm in acupoints located in limbs and abdomen without any manipulation like lifting and thrusting or twirling and rotating to avoid De qi sensation. The sham electrodes from the EA apparatus will be connected to the needle handles at non-acupoint 1 (NA1) and NA2. The sham electrode lines will be identical to the real one, but the inner metal wires will be cut off. Therefore, no current will be outputted with the EA apparatus in a power-on state with a lighted power indicator and voice of clatter. The parameters of sham EA apparatus and treatment will be the same as in the EA group. Participants will be treated individually to avoid communication.

**Table 2.** Locations of non-acupuncture points used in sham electroacupuncture group.

Non-acupoints	Location
NA1-2	3 cun lateral to the GB14 (On the forehead, 1 cun directly above the midpoint of the eyebrow) horizontally.
NA3-4	1 cun lateral to the CV15 (Below the xiphoid process, 7 cun above the umbilicus, locate the point in supine position with the arms uplifted) horizontally.
NA5-6	1 cun lateral to the CV12 (On the midline of the abdomen, 4 cun above the umbilicus) horizontally.
NA7-8	1 cun lateral to the CV9 (On the midline of the abdomen, 1 cun above the umbilicus) horizontally.
NA9-10	1 cun lateral to the CV5 (On the midline of the abdomen, 2 cun below the umbilicus) horizontally.
NA11-12	On the side of the abdomen, 2 cun above the anterior superior iliac spine.
NA13-14	1 cun lateral to the SJ6 (3 cun above SJ 4, between the radius and the ulna, on the radial side of muscle extensor digitorum) horizontally.
NA15-16	3 cun lateral to the LU6 (The point is on the palm aspect of the forearm, on the line joining Lu 9 and Lu 5, 7 cun above the transverse crease of the wrist) horizontally.
NA17-18	3 cun inferior to the GB34 (In the depression anterior and inferior to the head of the fibula) vertically.
NA19-20	1 cun inferior to the SP6 (3 cun directly above the tip of the medial malleolus, on the posterior border of the medial aspect of the tibia) vertically.

NA: Non-acupoint, GB14: Yangbai, CV15: Jiuwei, CV12: Zhongwan, CV9: Shuifen, CV5: Shimen, SJ6: Waiguan, LU6: Kongzui, GB34: Yanglingquan, SP6: Sanyinjiao.

#### 2.5.3. Permitted and prohibited concomitant treatments

All participants will be permitted to treat insomnia with zopiclone (7.5 mg/T, Guangdong Cr.shunfeng Pharmaceutical Co., Ltd., Guangdong, China, taken orally, 3.75 mg each time) as their first choice for acute medication during the trial. The name, dosage, and timing of usage will be recorded in the sleep diary.

### 2.6. Outcomes

#### 2.6.1. Primary outcome measurement

The primary is the response rate, which reflects the proportion of participants whose average Insomnia Severity Index (ISI) scores decrease by at least 7 units [30,31] at Week 4 compared with baseline. The ISI is a 7-item self-administrated instrument to evaluate the nature, severity, and impact of insomnia during daytime and nighttime. A 5-point Likert scale is used to score each item, consisting of a total score ranging from 0 to 28: where 0–7 = No clinically significant insomnia, 8–14 = Subthreshold insomnia, 15–21 = Clinical insomnia (moderate severity), 22–28 = Clinical insomnia (severe) [31,32]. The minimal clinically important difference (MCID) of ISI is 7 units [30,31].

#### 2.6.2. Secondary outcome measurement

Sleep diary: Participants will be required to complete a daily sleep diary during the whole trial, the variables in the daily sleep diary include sleep onset latency, wake after sleep onset, early morning awakening, total wake time, total sleep time, and sleep efficiency [31].
Insomnia Severity Index (ISI): ISI will also be used as one of the secondary outcomes, details of ISI are presented in primary outcome measurement section [31,32]. ISI will be applied at baseline, Week 4 and 8
Pittsburgh Sleep Quality Index (PSQI): PSQI will be used to assess sleep quality and disturbances over the past month. PSQI is composed of 19 items including 4 open questions, and 15 Likert-type questions that score for each question ranging from 0 to 3. Individual item scores yield 7 components. The final score is acquired by adding the 7 component scores. Higher scores indicate more acute sleep disturbances, and a cut-off score of 5 suggests poor sleep quality [33]. PSQI will be applied at baseline, Week 4 and 8.
Short Form Health Survey-12 (SF-12): SF-12 will be used to detect the quality of life. The SF-12 comprises both mental and physical components, with each part ranging between 0 and 100. Higher scores indicate a greater quality of life [34]. SF-12 will be applied at baseline, Week 4, and 8.
Fatigue Severity Scale (FSS): FSS is a 9-item questionnaire and will be used to assess the severity of fatigue and how it affects certain activities. Each item is scored on a seven-point scale, the total score ranges from 9 to 63. Higher scores indicate more severe fatigue [35]. FSS will be applied at baseline, Week 4, and 8.
(6) 24-Hour ambulatory blood pressure: 24-Hour ambulatory blood pressure monitoring will be applied as baseline, Week 4, and 8 to evaluate the night blood pressure, the results will be interpreted by a specific cardiovascular specialist.
Blood sample: Blood samples will be collected at baseline, Week 4, and 8 to compare the levels of C-reactive protein (CRP) and D-Dimer.

#### 2.6.3. Compliance assessment

Participants who achieve scores above 80 will meet the criteria for good compliance by the end of Week 4.

$$\text{Compliance score}=\frac{\text{Acutual treatment sessions}}{\text{Required treatment sessions}}x100$$

E.g. a participant undergoes 6 treatment sessions, as 12 sessions are required in this study, based on the equation, the compliance score amounts to 50, denoting inadequate compliance.

#### 2.6.4. Assessment of safety

All adverse events will be assessed and recorded by proficient acupuncturists and outcome assessors after the end of each treatment and during the follow-up phase. Serious adverse events will be reported and handled according to relevant regulations. Common adverse events associated with acupuncture include persistent post-needling pain, dizziness, subcutaneous hematoma, infection, etc.

### 2.7. Sample size

No previous study has been found to use the response rate to evaluate EA for insomnia in end-stage renal disease undergoing MHD. A formal sample size calculation is not necessary in our exploratory study. Thus, recruitment will take place for a participant sample size of 60, with 30 individuals per group, to obtain the practical aim of our study based on clinical experience [36].

### 2.8. Statistical analysis

A biostatistician, who is blinded to the data, is accountable for conducting statistical analyses employing SAS (SAS Institute located in Cary, NC, USA) and R software (version 4.2.1). Intention-to-treat (ITT) is our statistical strategy that includes all participants who have been randomized and received at least one intervention session. Demographic characteristics will be summarized for each group. Mean (standard deviation) or median (interquartile range) will be used to describe continuous variables depending on distribution’s normality. Percentage will be used to describe categorical variables. Regarding primary outcomes, a Chi-squared test will be applied to compare the response rate between treatment group and control group. For secondary outcomes, continuous variables including sleep diary, ISI, PSQI, SF-12, FSS, ambulatory blood pressure, and plasma levels of CRP and D-Dimer will be analyzed according to repeated measurement using a mixed-effects model. α will be set at 0.025 for the primary outcome, and it will be set at 0.05 for multiple comparisons of the secondary outcomes. Besides, the 95% confidence interval (95% CI) will be used to assess effect size of primary and secondary outcomes. A Chi-squared test will be applied for the compliance scores and adverse events rate. Last observation carried forward method will be used for the dropout analysis. Sensitivity analysis will be conducted using per-protocol (PP) analysis, which consists of all participants who meet all eligibility criteria and are randomized, complete the treatment and follow-up plan, without any major protocol deviations.

## 3. Discussion

Insomnia is regarded as a major concern and top priority to be addressed by patients with ESRD undergoing MHD due to multiple aetiological causes that involve medical, psychological and behavioral areas. Insomnia causes severe health problems and poor quality of life in patients with ESRD undergoing MHD, while it remains underrecognized and undertreated. Current treatments including CBT-I and drugs are unavailable for most patients or have limited evidence to prove the efficacy and safety. Therefore, alternative therapies are urgently needed. In China, EA is often used to treat insomnia, and a previous study [18] has confirmed its efficacy. However, a study [37] analyzed 2,293 participants with insomnia resulting from different medical conditions (stroke, ESRD, perimenopause, pregnancy, psychiatric diseases), claimed that current evidence is insufficient to support or refute acupuncture for treating insomnia due to methodological quality, high levels of heterogeneity and publication bias. Therefore, high-quality studies with well-designed methodology are crucial to assess the efficacy and safety of EA for insomnia in patients with ESRD undergoing MHD.

We cautiously designed the present study according to relevant guidelines, the study adheres to well-established methodological requirements, including adequate randomization, allocation concealment, and blinding of participants, outcome assessors and statisticians, providing reliable evidence on the efficacy and safety of EA for insomnia in ESRD undergoing MHD. Specifically, we will evaluate beneficial effects of EA on sleep onset latency, wake after sleep onset, early morning awakening, total wake time, total sleep time, and sleep efficiency. We will use computer-produced randomization and allocation concealment from a third party to minimize selection bias and apply block randomization to ensure a balanced prognosis between intervention groups. Participants, data analysts, and outcome assessors will be blinded to group allocation. The allocation of all participants will be concealed until the statistical analysis is completed. All processes follow traditional Chinese medicine theory and clinical practice. Due to the increased nocturnal systolic BPV and inflammatory factors induced by insomnia in ESRD patients, we will adopt outcome measurements including 24-Hour ambulatory blood pressure and blood sample (plasma levels of C-reactive protein (CRP) and D-Dimer) to objectively investigate the effect of EA for insomnia in ESRD undergoing MHD. There will be 3 sessions each week during the 4-week treatment period, resulting in a total of 12 sessions with a 4-week follow up. The well-designed study can provide a profound impact on the clinical practice of EA for insomnia in ESRD undergoing MHD.

There are several limitations in our study. First, the acupuncturists are not blinded. Therefore, acupuncturists may pay more attention to the participants in the treatment group, which may lead to the observed effects. Also, we use sham EA with false apparatus and superficial penetration on non-acupoints to isolate the specific effect of EA. Compared with placebo control, there is a possibility of having a therapeutic effect. However, we hypothesize that EA will have a better effect, so there is no need to set a placebo control group, and the sham control procedure has been successfully applied in previous study [29]. Moreover, our study is single-center, participants are from one hospital, which will cause limited representativeness of patients and poor extrapolation of our findings. However, single-center study has better internal authenticity compared with multi-center trials. Besides, the relatively small sample size may reduce the probability of rejecting a false null hypothesis, whereas, our study is a pilot study, a sample size of 30 for each group is recommended in previous studies. Finally, due to the treatment burden of patients with ESRD undergoing MHD, a fixed treatment schedule will not be set for everyone, we will suggest participants receiving treatment every other day, however, treatment schedule will be adjusted when overlapping MHD treatment.

In conclusion, we will conduct a single-center and two-arm RCT to investigate the efficacy and safety of EA for insomnia in ESRD undergoing MHD. We employ a strict methodology to minimize bias and form monitoring committees to guarantee the quality of the clinical trial, which provides evidence for further understanding and generalizing the clinical application of EA for insomnia in ESRD undergoing MHD.

## Data Availability

The CRFs will be preserved in a locked cabinet at the participating hospitals and can be accessed by the research team only. Patient identifiable data will be used to provide clinical care and follow-up merely, and the trial database will be anonymized. The aggregated research findings will be submitted for publication in a peer-reviewed clinical journal to have widespread dissemination. The original paper files and electronic data will be preserved for at least 5 years after publication, which can be accessible from the corresponding author with appropriate reasons.

## Reporting checklist

The authors have completed the SPIRIT reporting checklist.
